# Low-Cost Sensor System for Air Purification Process Evaluation

**DOI:** 10.3390/s24061769

**Published:** 2024-03-09

**Authors:** Arkadiusz Moskal, Wiktor Jagodowicz, Agata Penconek, Krzysztof Zaraska

**Affiliations:** 1 Faculty of Chemical and Process Engineering, Warsaw University of Technology, 00-645 Warsaw, Poland; wiktorjagodowicz@gmail.com; 2 Łukasiewicz Research Network—Institute of Microelectronics and Photonics, Kraków Division, 30-701 Kraków, Poland; krzysztof.zaraska@imif.lukasiewicz.gov.pl

**Keywords:** low-cost sensors, air purification, particle counters, gas sensors, volatile organic compound sensor

## Abstract

With the development of civilisation, the awareness of the impact of versatile aerosol particles on human health and the environment is growing. New advanced materials and techniques are needed to purify the air to reduce this impact. This brings the necessity of fast and low-cost devices to evaluate the air quality from particulate and gaseous impurities, especially in a place where gas chromatography (GC) techniques are unavailable. Small portable and low-cost systems may work separately or be incorporated into devices responsible for air-cleaning processes, such as filters, smoke adsorbers, or plasma air cleaners. Given the above, this study proposes utilising a self-assembled low-cost system to evaluate air quality, which can be used in many outdoor and indoor applications. ESP32 boards with the wireless communication protocol ESP-NOW were used as the framework of the system. The concentration of aerosol particles was measured using Alphasense sensors. The concentrations of the following gases were measured: NO_2_, SO_2_, O_3_, CO, CO_2_, and H_2_S. The system was used to evaluate the quality of air containing tobacco smoke after passing through an actual DBD plasma reactor where the purification occurred. A high amount of reduction in aerosol particles and a reduction in the SO_2_ concentration were detected. An increase in the NO_2_ concentration was seen as an undesirable effect. The aerosol particle measurements were compared with those using a professional device (GRIMM, Hamburg, Germany), which showed the same trends in aerosol particle behaviour. The obtained results are auspicious and are a step towards producing a low-cost, efficient system for evaluating air quality as well as indoor and outdoor conditions.

## 1. Introduction

The air quality we breathe is a matter of paramount concern for human health and environmental sustainability [1]. In the face of increasing urbanisation and industrialisation, air pollution has emerged as a global challenge, posing significant threats to public well-being and ecosystems. In response to this challenge, there is a pressing need to develop innovative air quality assessment and improvement technologies. A crucial aspect of this endeavour is the evaluation of air purification processes. Air pollution may become a global health crisis, affecting populations worldwide. It encompasses a broad range of airborne contaminants, including particulate matter (PM), volatile organic compounds (VOCs), nitrogen oxides (NOx), sulphur dioxide (SO_2_), carbon monoxide (CO), and various other hazardous substances. These pollutants originate from diverse sources, such as industrial processes, transportation, agricultural activities, and natural events [2]. Their presence in the atmosphere poses a significant risk to human health and the environment. Recent studies have highlighted the association between air pollution and various health issues, including respiratory and cardiovascular diseases and premature mortality [3,4].

Cigarette smoke is also an essential factor polluting the local air. Air purification from cigarette smoke presents a significant challenge due to the complex mixture of harmful compounds produced during tobacco combustion. Cigarette smoke contains various hazardous components, including particulate matter (PM), volatile organic compounds (VOCs), and toxic gases, which pose health risks to both smokers and non-smokers. Efforts to effectively purify air from cigarette smoke have led to the development of various innovative technologies. Cigarette smoke is composed of thousands of chemicals, and many of them are known carcinogens. It poses severe health risks, including an increased risk of lung cancer, respiratory diseases, and cardiovascular disorders [5,6]. Passive smoke exposure, also known as second-hand smoke, is especially detrimental, making air purification crucial in residential and public settings. A study by [7] examined the effectiveness of novel photocatalysis–plasma air purification technologies in removing particulate matter and VOCs from cigarette smoke. The research highlighted the limitations of standard air purifiers and suggested the need for specialised filtration systems to address the unique challenges tobacco smoke poses.

Air purification processes play a critical role in mitigating the effects of air pollution. These processes encompass various techniques and technologies, each designed to reduce the concentration of pollutants in the air. Standard air purification methods include mechanical filtration, chemical absorption, electrostatic precipitation, biological treatments, and advanced oxidation processes. Air purification using plasma reactors is a cutting-edge and promising technology that has gained significant attention in recent years. Plasma, often called the fourth state of matter, has unique properties that effectively eliminate airborne contaminants. This innovative approach holds the potential to revolutionise air purification systems and address the pressing global challenge of air pollution. Plasma reactors utilise high-energy electrical discharges to generate highly reactive and ionised gas, which can efficiently break down and neutralise various pollutants in the air. These pollutants may include volatile organic compounds (VOCs), odours, bacteria, viruses, and particulate matter. The reactive species produced in the plasma can interact with pollutants, leading to their degradation and removal. This method offers several advantages, such as a broad spectrum of pollutant removal, rapid kinetics, and minimal by-product formation. In a study [8], the authors explored the application of plasma technology to remove volatile organic compounds (VOCs) in indoor air. The research highlighted the effectiveness of non-thermal plasma reactors in decomposing VOCs, leading to improved indoor air quality. The review underlined the versatility of plasma technology in treating different types of pollutants and the environmental benefits it offers. Integrating plasma reactors into air purification systems showcases a promising pathway towards cleaner and healthier air. While challenges like energy efficiency and system scalability remain, ongoing research and development efforts continue to advance this transformative technology, making it an area of considerable scientific interest.

The advent of low-cost air quality sensors has introduced a paradigm shift in air quality monitoring. These sensors, characterized by their affordability, portability, and ease of deployment, have made high-resolution air quality data accessible to a broader audience. They can measure various air pollutants, including particulate matter (PM2.5 and PM10), volatile organic compounds (VOCs), carbon monoxide (CO), nitrogen dioxide (NO_2_), and ozone (O_3_), among others. Low-cost sensors have been applied in various settings, from research and citizen science initiatives to smart city infrastructural development [9,10,11,12]. Smart sensors can be used for the evaluation of indoor air quality, especially for measuring NO_2,_ O_3_, CO_2_, and CO concentrations. They are promising tools for air quality evaluations despite the lack of standardization of calibration, analysis procedures, evaluation of performance, handling, and quantification of interferences [13]. All these issues need deeper investigations.

This paper introduces an approach that harnesses low-cost sensors for evaluating air purification processes, especially those based on using non-thermal plasma, which have become popular for indoor air purification systems. It should be mentioned that non-thermal plasma generators have the ability to produce O_3_ and NO_2_ gases, which are hazardous to human health and may strongly affect air quality, so their concentrations must be carefully measured and monitored. Furthermore, the system is able to monitor the concentrations of other hazardous gases, e.g., CO and SO_2_, and can be used in all cases where the presence of these gases may occur, like in gaseous water heaters and coal stoves, to prevent dangerous concentrations of these gases in closed rooms.

This work aimed to design and build a measurement system for testing air quality using low-cost sensors to measure the concentrations of suspended dust and selected gases in the air. The motivation for making such a measurement system is to provide access to an inexpensive, quick, and easy-to-use tool for monitoring air purification processes, such as dedusting or the adsorption of gaseous pollutants on porous beds, as well as monitoring the air quality in the surroundings, which, due to its small size and mobility, could be used in various conditions to conduct preliminary research.

## 2. Materials and Methods

The system requirements were as follows:

- The possibility to measure the mass concentration of suspended dust fractions and particle size distribution in the tested air.
- The possibility to measure the temperature and relative humidity of the tested air.
- The possibility to measure gas concentrations (carbon monoxide, carbon dioxide, nitrogen dioxide, ammonia, hydrogen sulphide, sulphur dioxide, oxygen, ozone, and volatile organic compounds).
- The compatibility of measurement trends of meters in the measurement system with high-class reference meters.
- The recording and visualisation of measurement data in real time.
- Wireless communication and data transfer between devices in the measurement system.
- The mobility of the constructed measurement system.
- Low energy consumption and long operating time of the meters in the measurement system.
- Low cost of the entire measurement system compared with professional meters.

The air quality measuring system was designed to provide data on particulate matter and selected gaseous pollutant concentrations in the tested air using low-cost sensors. The system uses low-power and low-latency wireless data transfer that, combined with batteries for most sensors, provides good mobility, a long use time, and a near real-time preview of the measurement data. The mentioned advantages and low cost of the measuring elements make this system a good, cheap tool for preliminary research on the environment and various air purification technologies. The air quality measuring system consists of the following:

- Two identical optical particle counters (OPC-N3, Alphasense, Essex, UK) (named A and B);
- An electrochemical CO_2_ sensor (SEN0159, DFRobot, Shanghai, China);
- Seven electrochemical gas sensors for NO_2_, NH_3_, SO_2_, H_2_S, CO, O_2_, and O_3_ (series SEN04xx, DFRobot, Shanghai, China);
- A metal oxide volatile organic compounds (VOCs) sensor (SEN0394, DFRobot, Shanghai, China) using an SGP40 chip (Sensirion, Stäfa, Switzerland);
- A transmitter–receiver device used for the wireless control of the sensors and receiving measurement data;
- Data acquisition and visualisation software that saves and displays the charted data in near real time.

Each sensor is outfitted with the following listed electronic components:

- The Espressif Systems ESP-32 microcontroller platform is used for the general control of the measuring elements and wireless data transfer;
- An RGB LED indicator is used to indicate the state of the devices;
- In the case of low-power-consumption sensors (SEN04xx series gas sensors and VOC sensors), a 2200 mAh 3.7 V rechargeable battery serves as a power source and is accompanied by an Adafruit LC709302F battery gauge used for measuring the state of the battery;
- In the case of higher-power-consumption sensors (optical particle counters and CO_2_ sensors), DC sockets and 5 V 2.5 A AC adapter power electronics are added.

**Figure 1:**
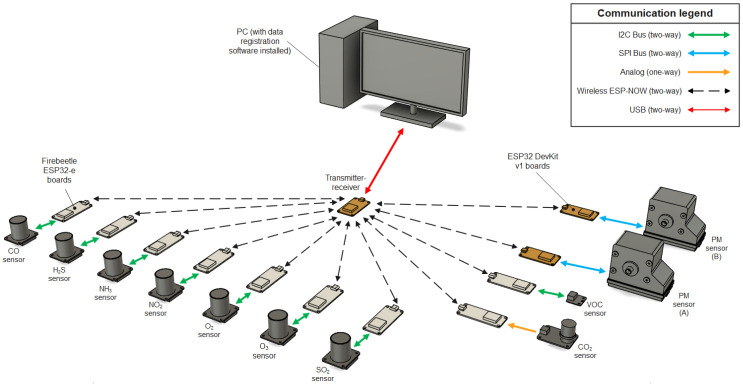
The system architecture.

The system architecture is presented in Figure 1.

**Figure 2:**
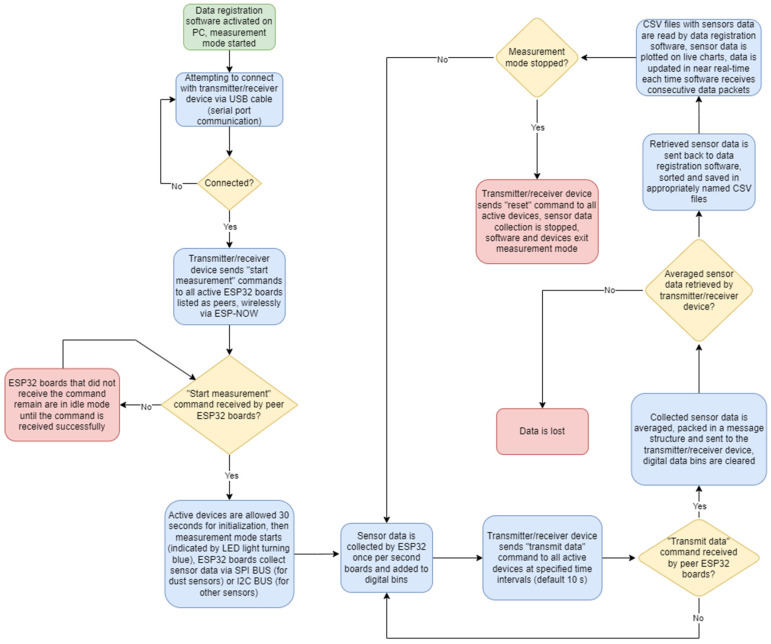
Explanation of how the measuring system works.

Each sensor is operated with an ESP32 microcontroller as well as the base transmitter–receiver unit connected to a computer. Communication between the sensors and transmitter–receiver unit is performed using the ESP-NOW communication protocol. The transmitter–receiver unit is connected to the computer using a standard USB connection and data transfer protocol. The electronics of all sensors and transmitter–receiver devices are housed in protective cases modelled using CAD software Autodesk Fusion 360 (v.2.0.16985) and were 3D-printed with fused deposition modelling (FDM) technology using PLA filaments. Figure 2 presents a step-by-step explanation of how the measuring system works.

After powering the sensors, the microcontrollers attempt to detect measuring elements and initialize various communication protocols depending on the sensor (I2C and SPI) and wireless communication protocol ESP-NOW. When the sensor initialisation is finished, the LED indicators turn green (if the measuring element is not detected by the microcontroller, the indicator will turn red instead). The sensors are then ready and waiting for commands from the transmitter–receiver device. After connecting the transmitter–receiver to the computer, a serial port communication is established using the included software. The software sends a data string to the transmitter–receiver via serial port communication. Upon receiving the appropriate data string, the transmitter–receiver broadcasts a command to all active sensors, ordering a measurement mode start. The command broadcast is realised using the ESP-NOW protocol. ESP-NOW is a wireless communication protocol defined by Espressif, which operates as a peer-to-peer (P2P) protocol, meaning it allows direct communication between two ESP8266 or ESP32 devices without the need for a central server or access point, e.g., a Wi-Fi^®^ router. Each ESP device has a unique MAC address that is used to identify the receiving board. Once all active sensors have started the measurement mode, the LED indicators turn blue, and the devices begin transmitting measurement data to the transmitter–receiver, relaying received data to the data acquisition software. The software takes care of sorting, organising, and saving the measurement data in appropriately named CSV files, which are then read by the software to display the saved data on charts that are updated in near real time, offering a preview of the measurement data collected while performing tests. Detailed information about the sensors is contained in Table 1. The manufacturer (DFRobot) does not provide detailed information on the internal workings of the sensors. The response time of the O_3_ sensor was verified by the authors, yielding a T90 value of about 90 s, which is in line with the manufacturer’s specifications. Typically, an electrochemical gas sensor works via the principle of electron exchange between the sensed gas and a layer of transition metal oxides. The sensor thus forms an electrochemical cell, and the output signal is proportional to the logarithm of the partial pressure of the sensed gas. The response typically follows the Nernst equation (see, e.g., [14], for a more detailed discussion of material and performance aspects). Since the sensor response is Nernstian, calculating the gas concentration requires temperature measurements, which are provided by the integrated temperature sensor. The manufacturer provides a software library [15] that performs these calculations automatically, using equations specific to each sensor type.

**Figure 3:**
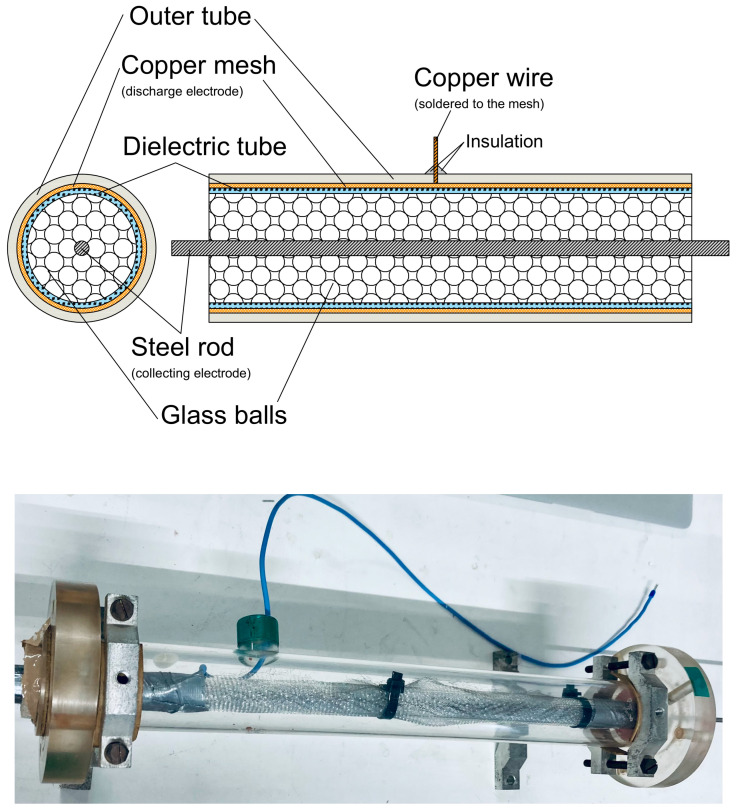
The plasma reactor.

To test our system, we chose cigarette smoke as a source of pollution. To reduce cigarette smoke, a DBD (dielectric barrier discharge) plasma reactor in its classical tubular geometry was designed, created, and tested during experiments. The reactor was based on a cylindrical dielectric tube made of PMMA with a diameter equal to 20 mm equipped with two electrodes (Figure 3). The outer electrode was made of copper wire mesh, and the inner one was made of a steel rod with a diameter equal to 5 mm. Between the electrodes, glass balls with a diameter of 3 mm were placed. This kind of plasma reactor was widely used in previous years [16].

The reactors were connected to an AC high-voltage generator designed and created in our laboratory. To monitor the power introduced into the reactor, an oscilloscope (54520A, HP, San Diego, CA, USA) equipped with an HV probe (HVP-18HF, Pintek Electronics, New Taipei City, Taiwan) connected to the outer electrode was used to measure the reactor voltage, while an 85 pF capacitor connected between the inner electrode and ground was used to measure the reactor charge. The time-averaged power ($\bar{P}$) introduced into the plasma was calculated using the following equation [17]:

(1) $$\bar{P}=\frac{1}{T}\int_{0}^{T}V\left(t\right)\cdot C_{m}\cdot \frac{dV_{m}}{dt}dt$$

where *V*(*t*) is the high voltage introduced into the system, *C_m_* is the capacitance of the measurement capacitor (85 pF), and *V_m_* is the voltage across the measurement capacitor.

Equation (1) shows the average power dissipated in a complete discharge cycle and may be evaluated by the area bounded by the Q–V diagram multiplied by the frequency of the discharge cycle. The maximum voltage generated in this system was at the level 20 kV_p-p_ and the power was about 50 W.

The experimental set-up presented in Figure 3 was used for air purification from cigarette smoke pollution. The smoke from cigarettes was sucked into tank 1 and introduced into the plasma reactor. After passing through the reactor, the air was sucked into tank 2, where gas sensors were placed. The flow through the system was established using an air pump (SC101MG0.2M, Emmecom, Singapore, Malaysia)) equipped with a valve that allowed the assumed airflow through the system. The airflow was measured using a flow meter (model 4001, TSI, Shoreview, MN, USA). The airflow through reactors during the experiments was established at 10 L/min. An aerosol spectrometer (model 1.109, GRIMM, Hamburg, Germany) was used to validate the results obtained from the aerosol particle meter sensors. A gas analyser (model GA-60, MADUR, Zgierz, Poland) was used to validate the NOx and CO concentrations in the air. To validate the O_3_ concentration measurements, an ozone meter (model OCT-3, DeltaTech Electronics, Jasło, Poland) was used.

## 3. Results and Discussion

### 3.1. Validation of Suspended Dust Meters

**Figure 4:**
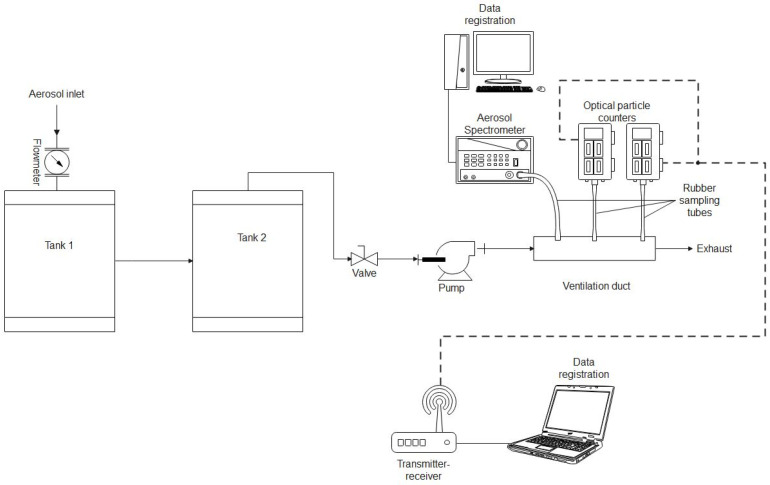
The experimental setup.

The meters were validated by comparing the measurements of the mass concentrations of suspended dust fractions obtained using the constructed meters (A and B) with the measurements obtained using the reference meter. Despite the ability of the built meters and the reference meter to measure the particle size distribution, no comparison was made in this respect due to differences in the measuring ranges of the meters (the built meters measured particles in the aerodynamic diameter range from 0.3 to 40.0 μm between 24 sorting channels, and the reference meter measured in the range 0.25–32.0 μm between 31 channels). Air polluted with smoke from burning matches was used as the research medium. This research was conducted using the research system shown in Figure 4. The airflow rate in the system during the test was approximately 5.5 L/min.

**Figure 5:**
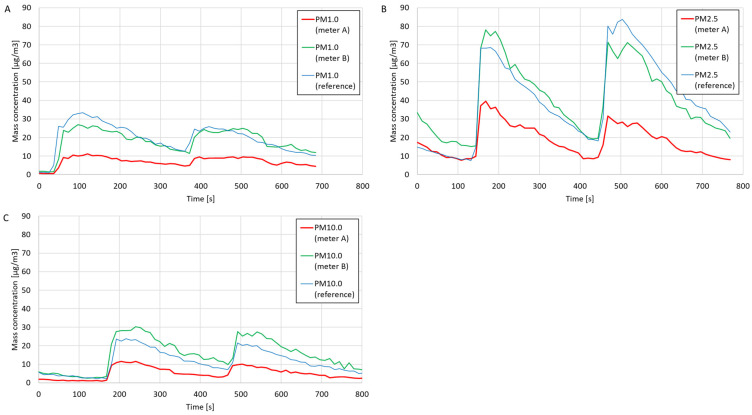
Mass concentrations of suspended dust fractions and validation of suspended dust meters: (**A**) PM1.0; (**B**) PM2.5; and (**C**) PM10.0.

The results of comparative measurements of the mass concentrations of PM1.0, PM2.5, and PM10.0 dust fractions are presented in Figure 5. After the measurements were performed, the average mass concentrations were calculated based on the dust meters and the reference meter measurements. The average percentage errors were determined by the readings of the reference meter. The determined average concentrations and percentage errors are presented in Table 2. Based on the values of the average percentage errors and comparing the curves on the charts, the compliance of the measurements and measurement trends of the constructed dust meters with the reference meter was assessed.

Based on the curves in the graphs in Figure 5, suitable agreement in the measurement trends was found between the constructed particulate matter meters and the reference meter. Based on the data in Table 2, poor agreement was seen regarding the measured average mass concentrations of PM1.0, PM2.5, and PM10.0 between the constructed meter A and the reference meter (the average percentage errors of all three measurement series were above 50%). In the case of meter B, high compliance was found with the average PM1.0 and PM2.5 concentration values obtained using the reference meter. The agreement between the PM10.0 concentration measurements using meter B and the reference meter was higher than that using meter A, but the percentage error was still high (−33.14%).

### 3.2. Validation of Gas Meters

**Figure 6:**
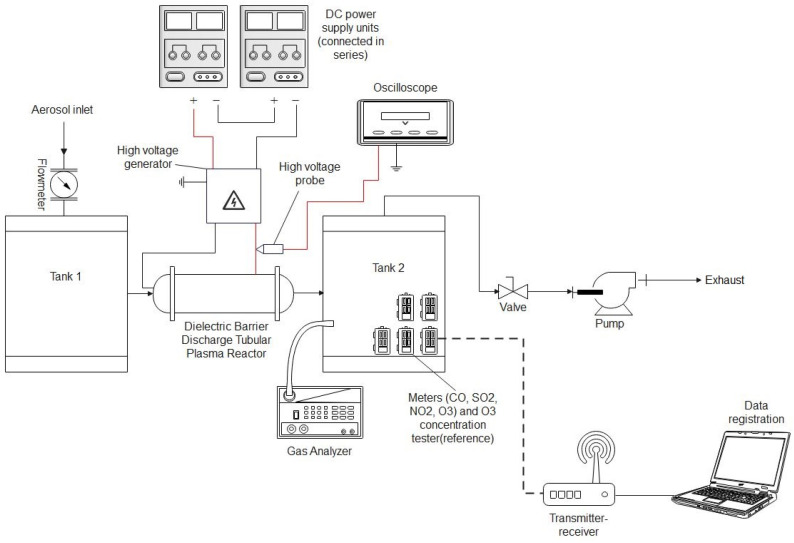
The experimental setup used for the validation of gas meters.

The meters were validated by comparing the gas concentration measurements obtained using built gas meters with measurements obtained using reference meters. Due to the lack of access to other reference meters, it was only possible to validate three of the nine built gas meters (validation was not carried out for the CO_2_, NH_3_, H_2_S, O_2_, SO_2_, and volatile organic compound meters). This research was carried out using the research system shown in Figure 6. During the validation of the CO meter, cigarette smoke was used as a gas source (in which, during previous tests, the presence of CO was detected using a built measurement system for air quality testing). During the validation of the NO_2_ and O_3_ meters, a previously tested prototype of a plasma reactor with barrier discharges was used as a gas source (during previous tests, its ability to generate both NO_2_ and O_3_ was demonstrated). During the operation, the airflow rate through the testing system was approximately 10 L/min. This research was carried out in two series. The first series was devoted to validating the CO meter, where cigarette smoke was introduced into the system, and the plasma reactor was not turned on. In the second series, simultaneous validation of the NO_2_ and O_3_ meters was carried out, where there was no cigarette smoke in the system, but the plasma reactor was turned on. The NO_2_ and O_3_ generation rate in the research system was controlled by regulating the voltage supplying the high-voltage generator connected to the reactor.

**Figure 7:**
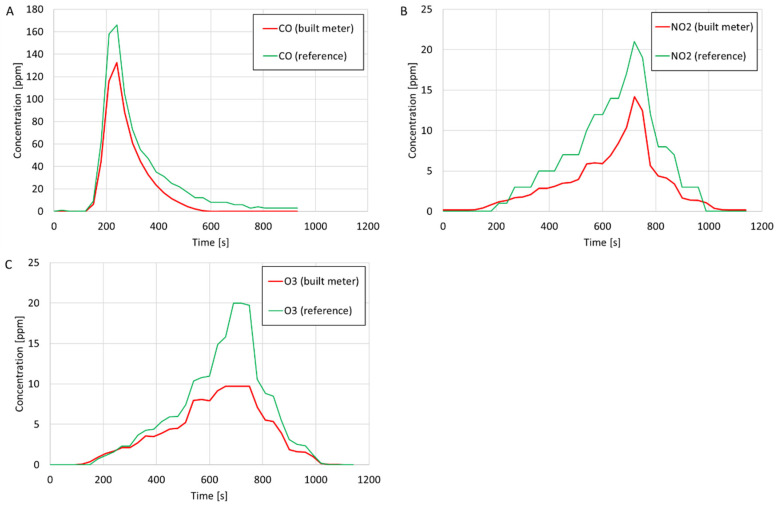
Validation of gas meters: (**A**) CO concentration (first series of measurements); (**B**) NO_2_ concentration (second series of measurements); and (**C**) O_3_ concentration (second series of measurements).

The results of comparative measurements of the volume concentrations of CO, NO_2_, and O_3_ are presented in Figure 7. After the measurements, the average gas concentrations were calculated based on measurements from the constructed and reference meters. The average percentage errors were determined in relation to the readings of the reference meters. The determined average concentrations and percentage errors are presented in Table 3. Based on the values of the average percentage errors and comparing the curves on the charts, the compliance of the measurements and measurement trends of the constructed gas meters with the reference meters was assessed.

Based on the curves in the charts in Figure 7A–C, good agreement between the measurement trends using the constructed CO, NO_2_, and O_3_ meters and the reference meters was found. Based on the average percentage errors presented in Table 3, relatively good agreement was found between the measured average CO, NO_2_, and O_3_ concentrations using the constructed and reference meters (the average percentage errors of the measurements amounted to, at most, 40.81%).

### 3.3. Ambient Air Quality Testing in the Laboratory

**Figure 8:**
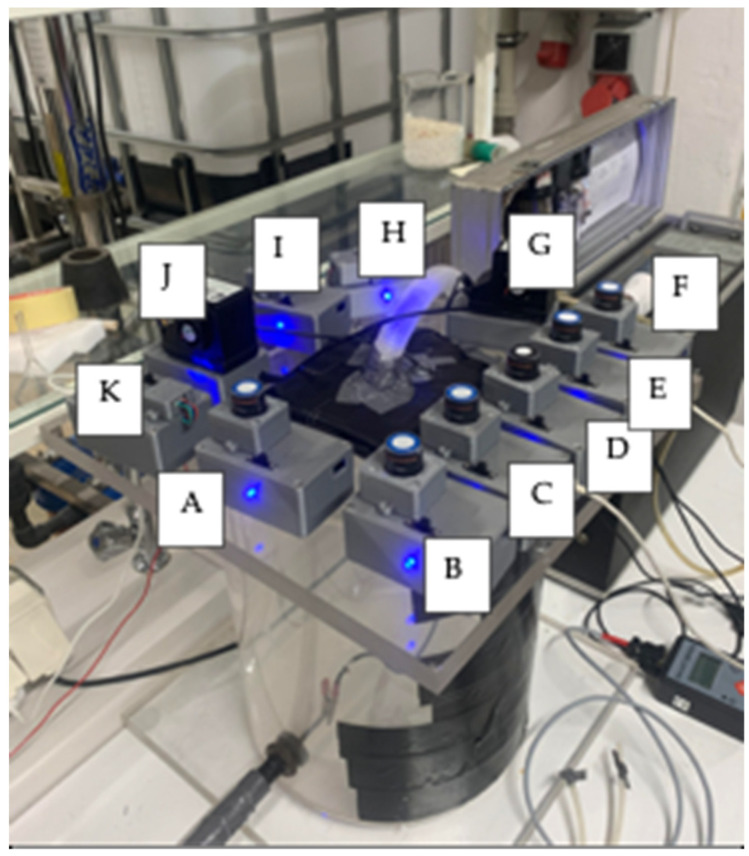
Measuring system to measure the quality of ambient air in the laboratory. A—CO senor, B—O_3_ sensor, C—H_2_S sensor, D—NH_3_ sensor, E—NO_2_ sensor, F—O_2_ sensor, G, J—AlphaSens particulate matter sensors, H—SO_2_ sensor, I—VOC sensor, and K—CO_2_ sensor.

The first test carried out using the constructed measuring system for air quality was an ambient air test in the laboratory. One suspended dust meter (B meter) and nine gas meters (CO, CO_2_, H_2_S, NH_3_, NO_2_, O_2_, O_3_, SO_2_, and VOC) included in the measurement system were used for this study. The arrangement of meters in the laboratory where the test was carried out is shown in Figure 8.

**Figure 9:**
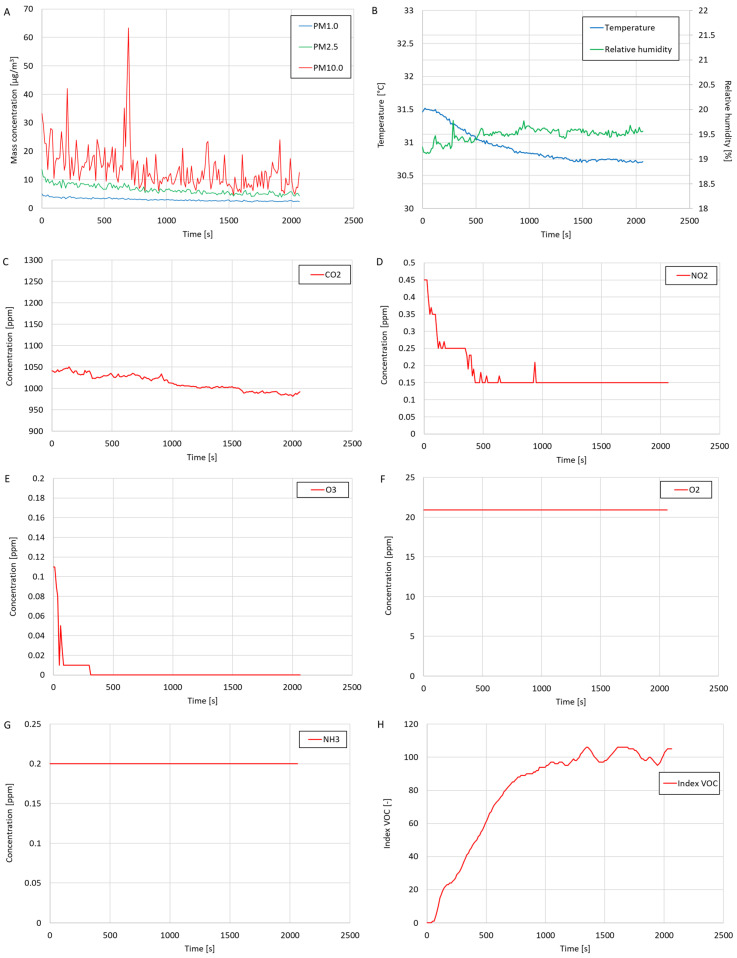
The test results of (**A**) mass concentrations of suspended dust fractions (PM1.0, PM2.5, and PM10.0); (**B**) temperature and relative humidity; (**C**) volume concentration of CO_2_; (**D**) volume concentration of NO_2_; (**E**) volume concentration of O_3_; (**F**) volume concentration of O_2_; (**G**) volume concentration of NH_3_; and (**H**) VOC index.

The test results are presented in the form of graphs of the mass concentration of suspended dust fractions (PM1.0, PM2.5, and PM10.0), volume concentrations of gases (CO_2_, NO_2_, O_3_, O_2_, and NH_3_), VOC index, temperature, and relative humidity in Figure 9. Charts of the CO, H_2_S and SO_2_ concentrations are not presented because these gases in the tested air were not detected during this study. Based on the test results, the average values of the measured values were calculated (as given in Table 4), and the maximum values were determined (as shown in Table 5).

Based on the test results, low mass concentrations of the suspended dust fractions PM1.0, PM2.5 and PM10.0 were found. The average CO_2_ concentration during the test was relatively high (1014 ppm), possibly because several people were present in the laboratory during the test (who exhaled CO_2_ into the surroundings). The oxygen concentration was constant during this study and amounted to 20.9%. During the test, trace amounts of NH_3_, NO_2_, and O_3_ were detected. The VOC index measured during this study was initially zero because the volatile organic compound meter was turned on only at the beginning of this study. The value of the VOC index increased over the duration of this study until, at the end of this study, the value oscillated at around 100. This means that for a longer part of this study, the volatile organic compound meter was calibrated against the current state of the quality of the tested air in terms of the content of volatile organic compounds. The oscillation of the VOC index value at around 100 means no significant changes in the concentration of volatile organic compounds in the tested air were detected during this study. The average temperature during this study was approximately 30.93 °C, and the average relative humidity was about 19.49%. The average temperature measured during this study was higher than the room temperature prevailing in the laboratory (approximately 25 °C, measured with a thermohydrometer), suggesting that the temperature measurements performed by the temperature sensor integrated into the OPC-N3 optical particle counter were overestimated. In the case of the average relative humidity, the value was lower than the value measured using the thermohydrometer by approximately 25%, which suggests that the relative humidity measurements by the OPC-N3 device were underestimated.

### 3.4. Testing the Quality of Air Polluted by Cigarette Smoke

**Figure 10:**
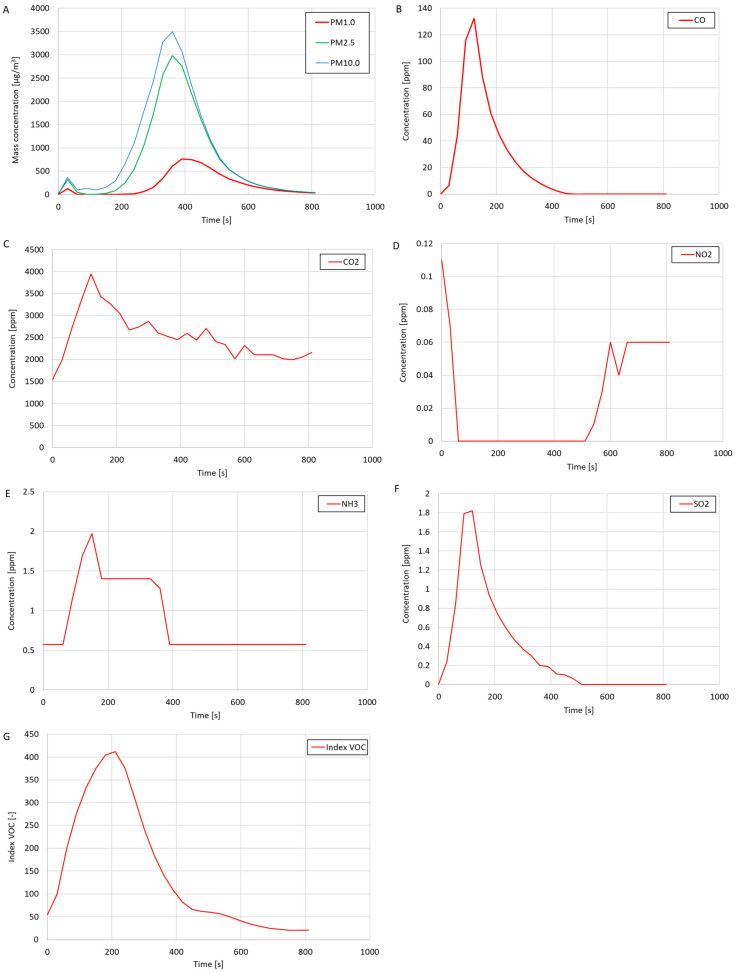
The results of testing the quality of air polluted by cigarette smoke: (**A**) mass concentration of suspended dust (dust meter B); (**B**) CO concentration; (**C**) CO_2_ concentration; (**D**) NO_2_ concentration; (**E**) NH_3_ concentration; (**F**) SO_2_ concentration; and (**G**) VOC index.

The study of the quality of air polluted by cigarette smoke was carried out in the test system shown in Figure 6. The smoke created by burning one cigarette was introduced into the system. The test results are presented in the form of graphs of the mass concentrations of suspended dust fractions (PM1.0, PM2.5, and PM10.0), the volume concentrations of gases (CO, CO_2_, NH_3_, NO_2_, O_2_, and SO_2_), and the VOC index in Figure 10. Graphs of the CO, H_2_S, O_3_, and SO_2_ concentrations are not presented because the presence of these gases in the tested air was not detected during this study. Based on the test results, the average values of the measured values were calculated (Table 6), and the maximum measured values were determined (Table 7). The test results concern the period from introducing the pollutant into the test system until the system was cleaned.

As a result of introducing cigarette smoke into the test system, significant increases in the mass concentrations of the suspended dust fractions PM1.0, PM2.5, and PM10.0, CO_2_ concentration, and NH_3_ concentration were observed, and a decrease in the NO_2_ concentration was regarded as the test system was filled with smoke. This study also showed the presence of CO (high concentration—max. 132.37 ppm) and SO_2_ (max. 1.82 ppm) in cigarette smoke. The oxygen concentration in the system was constant during the test and amounted to 20.9%. The measured VOC index increased with the introduction of smoke into the test system. The maximum measured VOC index value during the test was 412, which meant that introducing cigarette smoke into the system significantly increased the concentration of volatile organic compounds in the tested air. It was noticed that the measured mass concentration of the suspended dust fractions (Figure 10A) increased to its maximum value after a much longer time than the measured concentrations of CO, CO_2_, NH_3_, SO_2_, or the VOC index. It was also noticed that the maximum measured mass concentrations of PM2.5 and PM10.0 exceeded the measurement range of the OPC-N3 optical particle counter included in the suspended dust meter (the mass concentration measurement range was 0–2000 μg/m^3^). It was concluded that after introducing cigarette smoke into the test system, the concentration of dust particles was so high that the suspended dust meter showed incorrect readings. The electronics in the optical particle counter used could not keep up with the counting of particles in conditions of high dust concentrations. It is suspected that if the measuring range of the meter were more extensive, the course of the mass concentrations of suspended dust fractions would look different (it would probably be similar in shape to the course of the mass concentrations of dust fractions in the chart in Figure 9A). Considering the above observations and conclusions, it was concluded that the OPC-N3 optical particle counter is unsuitable for performing dust measurements in very high concentrations of suspended dust.

### 3.5. Testing the Quality of Air Polluted by Cigarette Smoke after Passing through a Plasma Reactor with Barrier Discharges

**Figure 11:**
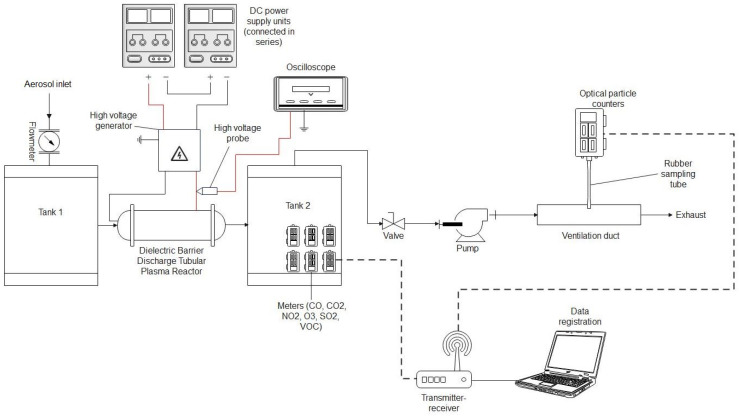
Diagram of the research system; testing of the plasma reactor with barrier discharges.

The test was carried out using the test system shown in Figure 11. The research system consisted of two cylindrical tanks. A prototype plasma reactor was installed between the outlet from tank 1 and the inlet to tank 2. Behind the outlet from tank 2, there was a pump sucking air into the testing system. The flow rate through the system was controlled by adjusting the opening degree of the ball valve installed at the pump inlet. The airflow rate through the test system was measured using a TSI 4000 series flowmeter. During the test, the flow rate was approximately 10 L/min. The high voltage applied to the electrodes in the plasma reactor was generated using a high-voltage generator powered by a pair of series-connected DC power supply units. This study involved introducing pollution into the research system in the form of cigarette smoke and checking the differences in air quality at the outlet of the plasma reactor before and after it was turned on. To measure the air quality during this study, one dust meter (B meter) and gas meters (CO_2_, CO, NO_2_, O_3_, SO_2_, and VOC) included in the air quality measurement system were used. The dust meter performed measurements at the outlet of the test system. Gas meters were placed in tank 2 for the duration of the test. The voltage applied to the electrodes in the plasma reactor was measured using an oscilloscope and a high-voltage probe.

**Figure 12:**
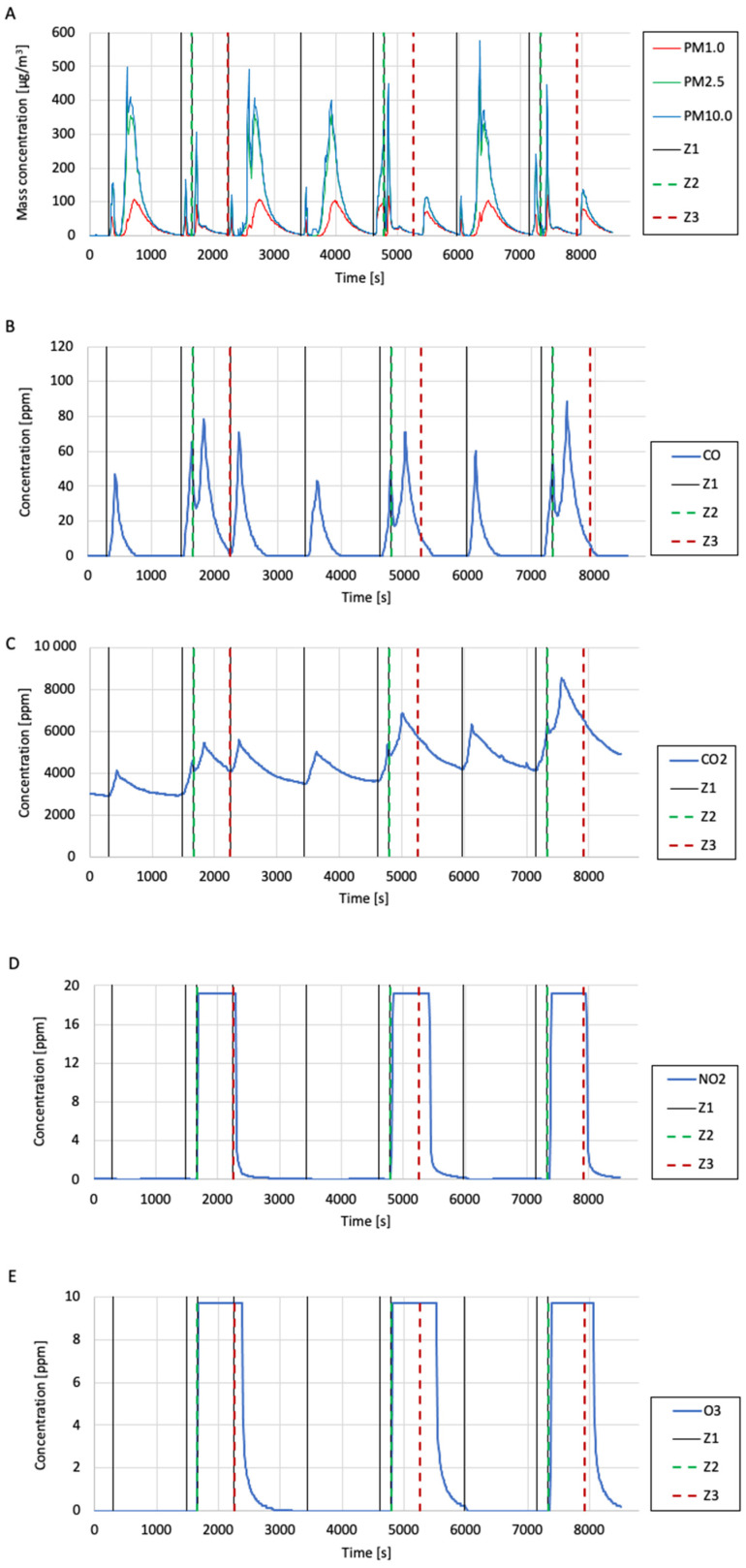

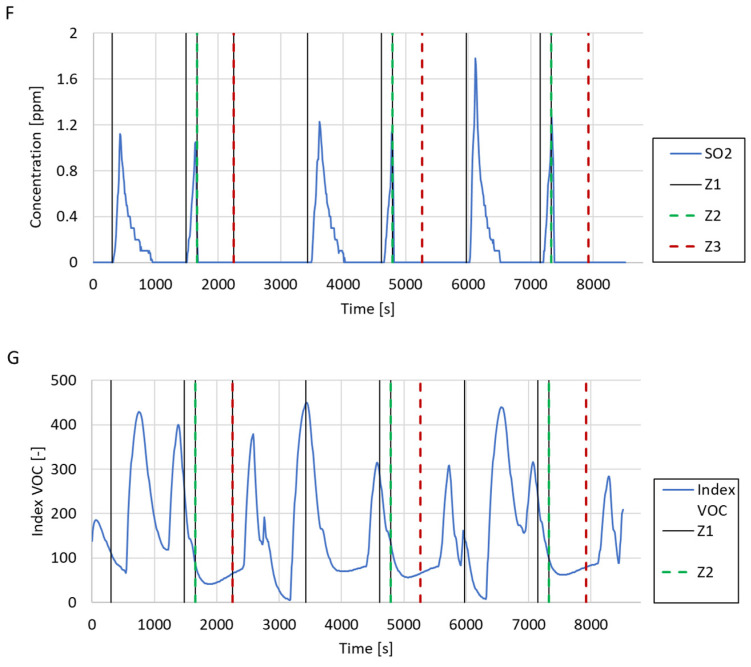
The results of testing the quality of air polluted by cigarette smoke after passing through the plasma reactor with barrier discharges: (**A**) mass concentration of suspended dust (dust meter B); (**B**) CO concentration; (**C**) CO_2_ concentration; (**D**) NO_2_ concentration; (**E**) O_3_ concentration; (**F**) SO_2_ concentration; and (**G**) VOC index.

The test results are presented in the form of graphs of the mass concentrations of suspended dust fractions (PM1.0, PM2.5, and PM10.0), volume concentrations of gases (CO_2_, CO, NO_2_, O_3_, and SO_2_), VOC index, temperature, and relative humidity in Figure 12. The graphs mark events such as introducing cigarette smoke (from burning one cigarette) into the research system (marked as “Z1”), turning on the plasma reactor (i.e., applying a high voltage, labelled as “Z2”), and turning off the reactor (marked as “Z3”) using vertical lines.

A similar trend was noticed in the graph of the mass concentration of the suspended dust fraction to that in the case of testing the quality of air polluted with cigarette smoke (the measurement results were erroneous for a certain period after the smoke was introduced into the system). However, it was observed that after the second and third shutdown of the plasma reactor (where, unlike the first shutdown, another portion of cigarette smoke was not introduced at the same time), the mass concentration of the suspended dust fraction rapidly increased, which suggests that during operation, the plasma reactor cleaned the air of solid particles, probably by the dedusting electrostatic mechanism. Analysing the CO concentration graph, it was observed that when cigarette smoke was introduced into the test system and the reactor was turned on, the CO concentration temporarily decreased and then increased. Based on this observation, there was a suspicion that the reactor may be able to decompose CO_2_ during operation. A similar situation was observed in the case of the CO_2_ concentration measurements. Based on the measurement results, it was found that the reactor generated significant amounts of NO_2_ and O_3_ during operation. Each time the reactor was turned on, the NO_2_ and O_3_ concentration values rapidly increased up to the upper limits of the measuring ranges of the meters used (measuring range of the NO_2_ meter—20 ppm; measuring range of the O_3_ meter—10 ppm). It is suspected that the amounts of NO_2_ and O_3_ generated by the reactor are much higher than the measurement ranges of the meters used. Based on the results of the SO_2_ concentration measurements, it was noticed that each time the reactor was turned on, the SO_2_ concentration in the tested air dropped almost immediately to 0 ppm, which suggests that the plasma reactor can decompose SO_2_ during operation. During this study, burning cigarettes did not achieve SO_2_ concentrations above approximately 1.8 ppm. Analysing the VOC index measurements performed during this study, it was observed that after each reactor switch-on, the VOC index value significantly decreased, even when subsequent cigarette smoke was simultaneously introduced into the test system. The VOC index value decreased until the reactor was shut down. After each reactor shutdown, the VOC index value increased again to very high values (between 400 and 450). This observation suggests that the plasma reactor can remove volatile organic compounds from the air during operation. Further research using professional equipment to measure the concentration of volatile organic compounds is recommended for verification.

## 4. Conclusions

During this investigation, building a measurement system for testing air quality using low-cost sensors was possible. According to the assumptions, this system is capable of measuring the mass concentrations of suspended dust fractions (excluding measurements in conditions of very high particle concentrations), particle size distribution, temperature, relative humidity, and the concentrations of selected gases, i.e., CO, CO_2_, NO_2_, NH_3_, H_2_S, SO_2_, O_2_, and O_3_. The ability to measure VOC concentrations could not be achieved due to the limitations of the used sensor. Instead of measuring concentrations, the measuring system can measure the relative changes in the content of volatile compounds in the tested air. These changes are presented in the form of the so-called VOC index. It should be mentioned that there are different sensors on the market with the same price range that can measure concentrations such as BME680, SGP30, and ENS160. Based on the validation tests of the meters included in the measurement system, it was established that the suspended dust meters (A and B) and the CO, NO_2_, and O_3_ meters are characterized by satisfactory compliance with measurement trends compared with high-class reference devices. Due to the lack of appropriate tools, it was impossible to determine the consistency of measurement trends between the CO_2_, NH_3_, H_2_S, O_2_, VOC meters and reference devices. Validation tests showed a complete lack of compliance with measurement trends between the built SO_2_ meter and a professional gas analyser. Due to the observations made during this study, more detailed research should be conducted in this direction. Validation tests of the dust meters included in the constructed measurement system showed that one of the meters, i.e., meter A, showed poor compliance with the reference meter in terms of the measured values of the mass concentrations of suspended dust fractions, while meter B showed high compliance. Due to the difficulties in using the CO_2_ meter for research, replacing the electrochemical CO_2_ sensor contained in the meter with an NDIR sensor is proposed. The Python application enables the control of the measurement system and the recording and visualization of the measurement data in near real time. The use of development boards with ESP32 microcontrollers and the implementation of the ESP-NOW communication protocol in the software of the devices included in the measurement system enables wireless communication and data transfer between the devices. The compact size of the constructed meters and the use of miniature batteries to power most of the meters ensure the mobility of the measurement system. Most electronic components selected for the built meters in the system are characterized by low energy consumption, which provides long operation times for the meters in the measuring system. The requirement of the low cost of the entire measurement system of professional meters was met. The total cost of building the measurement system was less than USD 2500.

## Figures and Tables

**Table 1:** Information about sensors (accessed on 30 January 2024).

Sensor	Manufacturer	Model	T90	Range	URL	Principle of Measurement	Detailed Specification
CO	DFRobot	SEN0466	≤30 s	0–1000 ppm	https://www.dfrobot.com/product-2508.html	Electrochemical -lack detailed information about specific chemical reactions	https://dfimg.dfrobot.com/nobody/wiki/5953b463b8712f03d0791e98dd592e78.pdf https://wiki.dfrobot.com/SKU_SEN0465toSEN0476_Gravity_Gas_Sensor_Calibrated_I2C_UART
NO_2_	DFRobot	SEN0471	≤30 s	0–20 ppm	https://www.dfrobot.com/product-2515.html		
NH_3_	DFRobot	SEN0469	≤150 s	0–100 ppm	https://www.dfrobot.com/product-2513.html		
SO_2_	DFRobot	SEN0470	≤30 s	0–20 ppm	https://www.dfrobot.com/product-2514.html		
H_2_S	DFRobot	SEN0467	≤30 s	0–100 ppm	https://www.dfrobot.com/product-2511.html		
O_2_	DFRobot	SEN0465	≤15 s	0–25% vol	https://www.dfrobot.com/product-2510.html		
O_3_	DFRobot	SEN0472	≤120 s	0–20 ppm	https://www.dfrobot.com/product-2516.html		
VOC	DFRobot	SEN0394	≤30 s	1–500 VOC index	https://www.dfrobot.com/product-2251.html	MOX Sens (patent pending Sensirion)	https://dfimg.dfrobot.com/nobody/wiki/e0d14f074941edff71a2368e8ee2ee76.pdf
CO_2_	DFRobot	SEN0159	≤3 s	350–10,000 ppm	https://www.dfrobot.com/product-1023.html	Electrochemical -lack detailed information about specific chemical reactions	https://image.dfrobot.com/image/data/SEN0159/CO2b%20MG811%20datasheet.pdf
PM	AlphaSense	OPC-N3	≤3 s	0.35–40 μm; 0–2000 μg/m^3^	https://www.alphasense.com/products/optical-particle-counter/	Optical	https://www.alphasense.com/wp-content/uploads/2022/09/Alphasense_OPC-N3_datasheet.pdf

**Table 2:** The determined average mass concentrations and percentage errors; validation of suspended dust meters.

	Average Value		
Sensors	A	B	Reference
PM1.0 [μg/m^3^]	7.15	18.27	19.76
Percentage errors (PM1.0) [%]	63.82	7.53	
PM2.5 [μg/m^3^]	18.20	40.73	40.09
Percentage errors (PM2.5) [%]	54.61	−1.60	
PM10.0 [μg/m^3^]	5.14	14.97	11.24
Percentage errors (PM10.0) [%]	54.27	−33.14	

**Table 3:** The average concentrations and percentage errors; validation of gas meters.

	Average Value	
	Gas Meters	Reference
CO [ppm]	18.49	27.75
Percentage error (CO) [%]	33.38	
NO_2_ [ppm]	3.19	5.38
Percentage error (NO_2_) [%]	40.81	
O_3_ [ppm]	3.51	5.39
Percentage error (O_3_) [%]	34.94	

**Table 4:** Average values of measured values; air quality testing in the laboratory.

Average Value											
Concentration											VOC Index [-]
Mass			Volume								
PM1.0 [μg/m^3^]	PM2.5 [μg/m^3^]	PM10.0 [μg/m^3^]	CO_2_ [ppm]	CO [ppm]	H_2_S [ppm]	NH_3_ [ppm]	NO_2_ [ppm]	O_2_ [%]	O_3_ [ppm]	SO_2_ [ppm]	
3.35	7.01	14.97	1014.00	0.00	0.00	0.20	0.18	20.90	0.0036	0.00	78.92

**Table 5:** Maximum values of measured values; air quality testing in the laboratory.

Maximum Value											
Concentration											VOC Index [-]
Mass			Volume								
PM1.0 [μg/m^3^]	PM2.5 [μg/m^3^]	PM10.0 [μg/m^3^]	CO_2_ [ppm]	CO [ppm]	H_2_S [ppm]	NH_3_ [ppm]	NO_2_ [ppm]	O_2_ [%]	O_3_ [ppm]	SO_2_ [ppm]	
4.91	12.20	67.44	1050.50	0.00	0.00	0.20	0.45	20.90	0.11	0.00	106.00

**Table 6:** The average values from testing the quality of air polluted by cigarette smoke.

Average Value											
Concentration											VOC Index [-]
Mass			Volume								
PM1.0 [μg/m^3^]	PM2.5 [μg/m^3^]	PM10.0 [μg/m^3^]	CO_2_ [ppm]	CO [ppm]	H_2_S [ppm]	NH_3_ [ppm]	NO_2_ [ppm]	O_2_ [%]	O_3_ [ppm]	SO_2_ [ppm]	
209.40	712.21	890.08	2517.68	21.13	0.00	0.88	0.02	20.90	0.00	0.36	146.79

**Table 7:** The maximum values from testing the quality of air polluted by cigarette smoke.

Maximum Value											
Concentration											VOC Index [-]
Mass			Volume								
PM1.0 [μg/m^3^]	PM2.5 [μg/m^3^]	PM10.0 [μg/m^3^]	CO_2_ [ppm]	CO [ppm]	H_2_S [ppm]	NH_3_ [ppm]	NO_2_ [ppm]	O_2_ [%]	O_3_ [ppm]	SO_2_ [ppm]	
760.38	2978.15	3493.55	3947.00	132.37	0.00	1.97	0.11	20.90	0.00	1.82	412.00

## Data Availability

Data are contained within the article.
